# Summary of discordant results between rapid diagnosis tests, microscopy, and polymerase chain reaction for detecting *Plasmodium* mixed infection: a systematic review and meta-analysis

**DOI:** 10.1038/s41598-020-69647-y

**Published:** 2020-07-29

**Authors:** Manas Kotepui, Kwuntida Uthaisar Kotepui, Giovanni De Jesus Milanez, Frederick Ramirez Masangkay

**Affiliations:** 1 Medical Technology, School of Allied Health Sciences, Walailak University, Tha Sala, Nakhon Si Thammarat, Thailand; 2 Department of Medical Technology, Institute of Arts and Sciences, Far Eastern University-Manila, Manila, Philippines

**Keywords:** Epidemiology, Diagnostic markers, Infectious diseases

## Abstract

Malaria rapid diagnostic tests (RDTs) are widely used to detect malaria parasites among patients who suspected malaria infections in malaria-endemic areas where microscopy is unavailable. Nevertheless, little is known about the performance of RDTs in detecting *Plasmodium* mixed infections. The present study aimed to evaluate the discordant results between RDTs and microscopy/polymerase chain reaction (PCR) in detecting *Plasmodium* mixed infections. The PubMed (MEDLINE), Web of Science, and Scopus databases were systematically reviewed to identify related studies that reported the performance of RDTs in detecting *Plasmodium* mixed infections**.** Studies were grouped according to the different RDT types including RDT type 2 (pf-HRP2/pan-aldolase), RDT type 3 (pf-HRP2/pan-pLDH), RDT type 4 (Pf-LDH/pan-pLDH), RDT type 5 (Pf/Pv-pLDH), and RDT type 6 (pf-HRP2/Pv-pLDH) for subgroup analysis. The estimates of the different proportions in each analysis group that were visually summarized in a forest plot showed the odds ratio (OR) and 95% confidence interval (CI). Plots were drawn using RevMan (version 5.3; Cochrane Community). Twenty-eight studies were included in the present study. Overall, the meta-analysis showed that RDTs could detect a significantly higher proportion of *Plasmodium* mixed infections than microscopy (p = 0.0007, OR = 3.33, 95% CI 1.66–6.68). Subgroup analysis demonstrated that only RDTs targeting Pf-specific histidine-rich protein 2 (HRP2)/pan-specific lactate dehydrogenase (LDH) could detect a significantly higher proportion of *Plasmodium* mixed infections than microscopy (p = 0.004, OR = 8.46, 95% CI 2.75–26.1). The subgroup analysis between RDTs and PCR methods demonstrated that RDTs targeting Pf-specific HRP2/Pv-specific LDH could detect a significantly lower proportion of *Plasmodium* mixed infections than PCR methods (p = 0.0005, OR = 0.42, 95% CI 0.26–0.68). This is the first study to summarize the discordant results between RDTs and microscopy/PCR in detecting *Plasmodium* mixed infections. Malaria RDTs targeting Pf-HRP2/pan-pLDH could detect a higher proportion of *Plasmodium* mixed infections than microscopy, while RDTs targeting Pf-HRP2/Pv-specific LDH could detect a lower proportion of *Plasmodium* mixed infections than PCR methods. The results of this study will support the selection and careful interpretations of RDTs for a better diagnosis of *Plasmodium* mixed-species infections and appropriate treatment of malaria patients in endemic and non-endemic settings.

## Introduction

Malaria is a public health problem reported worldwide especially in the African region (213 million or 93%), with an estimated 405,000 deaths from malaria globally in the year 2018^1^. Human malaria is caused by five species of *Plasmodium* spp. including *P. falciparum*, *P. vivax*, *P. malariae*, *P. ovale*, and *P. knowlesi*^2^. Microscopy and rapid diagnostic tests (RDTs) are diagnostic tools to confirm the diagnosis in patients suspected of having malaria^1^. Currently, the microscopic method is the gold standard for malaria detection and diagnosis. However, it is imperfect by nature, especially in the identification of mixed-infections among residents in community-endemic areas. Sub-microscopic mixed-infections with low parasite density are commonly missed by microscopic methodologies^3^. Therefore, mixed infections of *Plasmodium* spp. are often unrecognized and underestimated due to the low detection rate by microscopy (2%)^4,5^. Misdiagnosis of *Plasmodium* mixed infections can lead to anti-malarial drug resistance and the development of severe malaria^6^. RDTs are easy to use and cost effective. They play a crucial role in the control of malaria when microscopy is unavailable and are convenient to use in field surveys or remote areas where laboratory capacity is limited. RDTs are immunochromatographic lateral flow devices of which commonly targeting histidine-rich protein-2 (HRP2), lactate dehydrogenase (LDH), and aldolase RDTs for rapid malaria detection^7–10^. RDTs targeting HRP2 are specific for the detection of *P. falciparum*, while RDTs targeting LDH can be used for the detection of *P. falciparum*, *P. vivax*, or pan-specific (e.g., four *Plasmodium* species) LDH antibodies; aldolase is another common target for RDTs to detect all *Plasmodium* species^7–10^. Recently used commercial dipsticks for the detection of HRP-2 include PfHRP2 CareStart^11–13^, SD Bioline Malaria Ag Pf^14,15^, and SD BIOLINE Malaria Ag P.f/Pan^16^^,^ and one recently used for the detection of pLDH is CareStart pLDH(pan)^15^. A recently used commercial dipstick for the detection of Pan-aldolase is ParaHit Total, while a recently used commercial dipsticks for the detection of *P. vivax* aldolase is mAb 1C3-12 F10^17^. Recently used commercial dipsticks for the detection of HRP-2/pLDH include SD BIOLINE Malaria Ag P.f/Pan^16^ and CareStart malaria HRP2/pLDH (Pf/pan) Combo test^18^. Finally, recently used commercial dipsticks for the detection of HRP-2/pan-aldolase include Malaria P.f/Pan Rapid Test Device Acon^19^ and ParaHIT Total Dipstick^20^.

Even though a large number of RDTs are available for malaria detection, the widespread use of RDTs causes the missed detection of mixed-species infections in individuals^21^. Moreover, their performance for the detection of mixed-species infections is less requires well more comprehensive studies. Since the accurate detection of mixed-species infections of malaria is very critical for successful malaria control programmes, the objective of this systematic review and meta-analysis was to summarise and analyse the performance of malaria RDTs in detecting *Plasmodium* mixed infections. This study aims to highlight the big knowledge gap on the performance of malaria RDTs in detecting these mixed-species infections and to help make informed decisions on the use of RDTs for prompt treatment, which will help eliminate malaria in endemic and non-endemic areas.

## Methods

### Search strategy

Searches of Medline (PubMed), Web of Science, and Scopus were systematically performed using the search terms provided in Supplementary Table S1. The searches were limited to the English language. Searches were carried out and finished on 1 April 2020. All reference lists of all eligible and included studies as well as Google Scholar search was performed to further increase the number of included articles for review.

### Definition of malaria RDTs and microscopy

Types of malaria RDTs were classified according to the study by Bell et al.^7^. They classified malaria RDTs into seven types according to the antigen used in the reagent strip, including type 1 (HRP2 (falciparum-specific), 2 (pf-HRP2/pan-aldolase), 3 (pf-HRP2/pan-pLDH), 4 (Pf-LDH/pan-pLDH), 5 (Pf/Pv –pLDH), 6 (pf-HRP2/Pv-pLDH), and 7 (aldolase). RDTs types 2, 3, 4, 5 and 6 can detect mixed or concurrent infections. Interpretation of *Plasmodium* mixed-infections by RDT was based on details provided by authors of the included studies. The gold standard for malaria detection is still microscopy where the examinations of thin and thick blood films lead to the demonstration of malaria parasites.

### Inclusion and exclusion criteria

Cross-sectional studies that reported the number of *Plasmodium* mixed infections evaluated by any of the five types of RDTs (types 2, 3, 4, 5 and 6) in comparison to microscopy or PCR were included in the present study. Studies reporting the results of RDTs and microscopy from the same patient samples or those reporting the results of RDTs and PCR from the same patient samples were included in the study. The following types of literature were excluded; studies that reported mixed-infections only for RDTs but did not report microscopy or PCR, incomplete data, no RDT results, co-infections with other agents, experimental studies, review articles, case reports and case series, polymorphism/mutation studies, knowledge about malaria/practice assessments, animal/mosquito studies, studies of haematological alterations, guidelines, and clinical drug trials. Studies with no full text and present data in the local language were also excluded.

### Data extraction

All studies acquired through the search were stored in EndNote reference manager software (version X9; Clarivate Analytics). The data extractions started with screening the titles and abstracts after duplicate studies removed. Studies that were not related to the inclusion criteria were excluded. Then, the studies were screened for full-text articles, and those that did not comply with eligibility criteria were excluded with tags indicating the reason for exclusion. The data from full-text articles that passed the inclusion and exclusion criteria were then exclusively examined and extracted by two independent authors (MK and KUK) using an Excel spreadsheet for further analysis. Any inconsistencies relating to included studies and data extraction were resolved by a third or a fourth reviewer (FRM or GDM).

### Statistical analysis

Studies were grouped (subgroup) according to the different RDT types for comparative analysis. The meta-analysis of the proportion of the number of *Plasmodium* mixed infections per the total number of total malaria positives were performed as follows: (1) the summary estimate of the difference in the proportion (odds ratios, ORs) of RDTs to detect mixed infections compared with microscopy and (2) the summary estimate of the difference in the proportion (ORs) of RDTs to detect mixed infections compared with PCR methods were estimated. The subgroup analysis of RDT types, blood collection methods (finger prick or venipuncture), and types of *Plasmodium* mixed species confirmed by PCR were analysed in the present study. All analyses were conducted using Review Manager Version 5.3 (Cochrane, UK). The statistical analysis used to calculate the difference between groups was the Mantel–Haenszel test with a random-effects model. The meta-analysis for each study and the overall studies are presented with OR and 95% confidence intervals (CIs) as effect measures and summarized in forest plots. Cochrane’s Q test and Higgins’s I^2^ statistics were performed to assess the heterogeneity of the included studies.

### Quality of included studies

The quality of the individual studies included in the present study was assessed by the Quality Assessment of Diagnostic Accuracy Studies (QUADAS)^22^. The tool includes 4 domains including the following: (1) report the review question, (2) develop review-specific guidance, (3) review the published flow diagram, and (4) judge bias and applicability. Each domain was assessed in terms of the patient selection, index test, reference standard, and flow timing. Patient selection was the method of patient selection reported in the included studies. The index test was the RDT method that was conducted and interpreted in the included studies. The reference standards were microscopy or the PCR method that was conducted and interpreted. The flow and timing described any patients who did not receive the index tests or reference standard. Each question was answered with a “yes,” “no,” or “unclear” response. The results of the QUADAS assessment for all included studies were then summarized in the methodological quality graph and summary created by Review Manager.

### Publication bias

Publication bias is the publication of studies due to the statistical significance of the results^23^, which can lead to overestimated effect sizes and the dissemination of false-positive results^24^. The publication bias was assessed by visual inspection of funnel plot asymmetry (the asymmetrical distribution of the included studies in the graph between the OR and SE (logOR)). The publication bias was also assessed with Egger's test. Both tests aimed to determine small-study effects leading to more or less beneficial summaries of OR estimates^25^.

## Results

### Characteristics of the included studies

**Figure 1:**
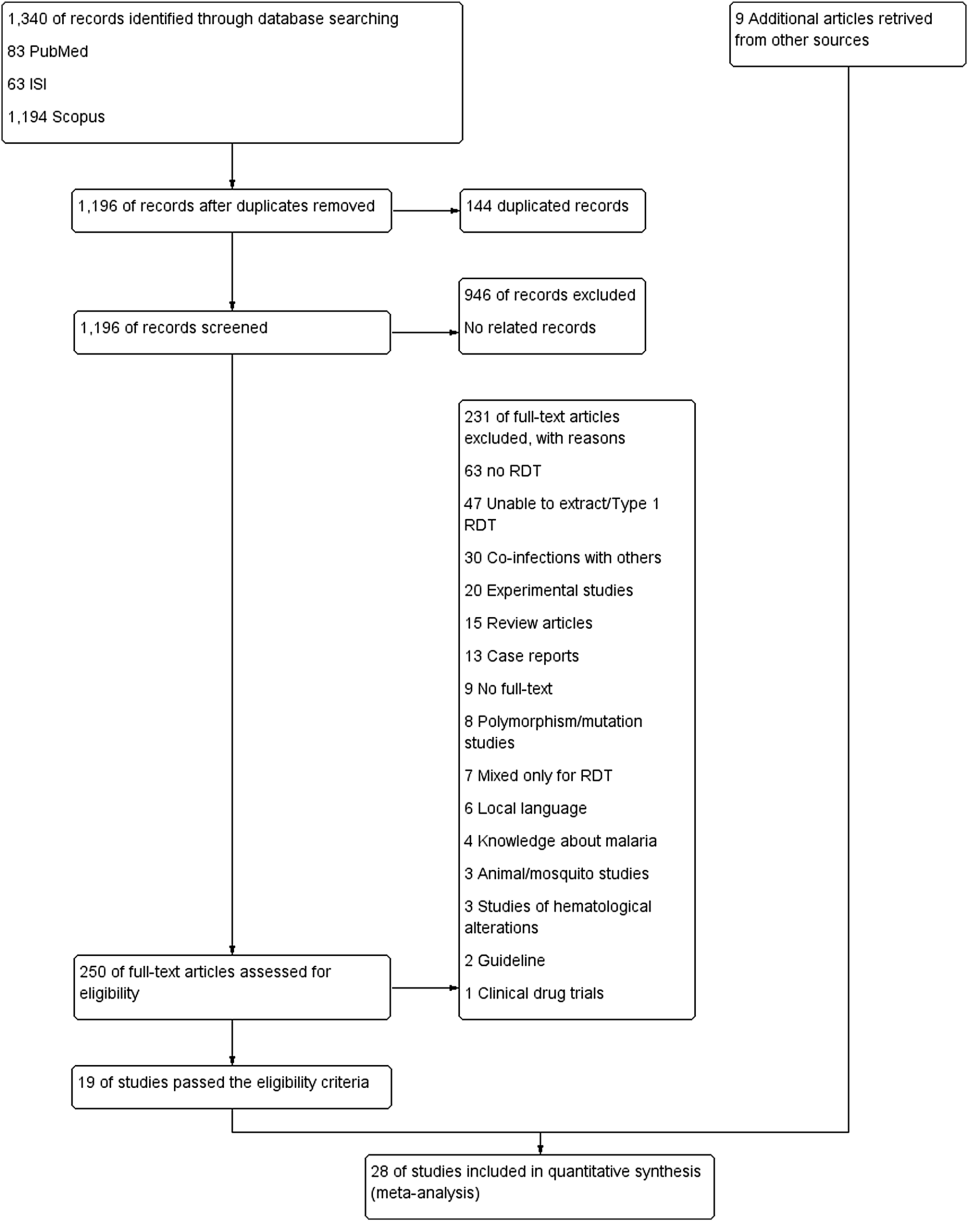
Study flow diagram.

The search retrieved 1,340 records. After removing 144 duplicates, 1,196 records were left for the title and abstract screening. Title and abstract screening resulted in the exclusion of 946 records. The full texts of 250 articles were assessed for their eligibility, and 231 of these were excluded with tags indicating the reason for exclusion. The most common reason for exclusion was no report of RDT in their articles. Other reasons for exclusion are shown in Fig. 1. As a result, 19 articles were included in the present study^26–44^. Further searches on the references of the selected publications which passed the inclusion criteria and Google Scholar search resulted in the inclusion of 9 additional articles^19,21,45–51^. Overall, 28 articles were selected, extracted, and analysed.

Among the 28 articles included in the present study, 3 reported mixed infections by RDT type 2 (pf-HRP2/pan-aldolase) and microscopy^27,41,51^, 13 by RDT type 3 (pf-HRP2/pan-pLDH) and microscopy^19,21,27,28,30,31,33,35,38,40,43,44,47^, 3 by type 4 (Pf-LDH/pan-pLDH) and microscopy^34,36,51^, 1 by RDT type 5 (Pf/Pv-pLDH)^46^, and 9 by RDT type 6 (pf-HRP2/Pv-pLDH)^21,26,36,42,43,46,48–50^. Among 27 articles included in the present study, 1 reported mixed infections by RDT type 2 and PCR^32^, 5 on RDTs type 3 and PCR ^21,28,33,43,45^ and 5 by RDT type 6 and PCR^21,26,29,37,43^. Most of the included studies (8/26, 30.8%) were conducted in Ethiopia^27,30,31,38,42,45,48,50^, India (3/26, 11.5%)^32,39,49^, and Kuwait (2/26, 7.7%)^34,46^. Additional data are shown in Table 1.

**Table 1:** Characteristics of the included studies.

No. (Ref.)	Author	Study area (years of the survey)	Participants (N)	Microscopy				RDTs						Molecular techniques			
				Malaria positive	Mono infections	Mixed infections	Method	Manufacturers	Antigen	Type	Malaria positive	2Mono infections	Mixed infections	PCR method (gene)	Malaria positive	Mono infections	Mixed infections
1. Ref.^26^	Alam et al., 2011	Bangladesh (2009–2010)	Febrile patients (338)	189	186	3	Thick and thin blood films	Onsite Pf/Pv (CTK Biotech Inc, USA)	pf-HRP2/Pv-pLDH	6	178	173	5^d^	Real-time PCR (18S rRNA)	188	180	8
								FalciVax Pf (Zephyr Biomedicals, India)	pf-HRP2/Pv-pLDH	6	191	189	2^d^				
2. Ref.^27^	Ashton et al., 2010	Ethiopia (2009)	Febrile patients (2,383)	552	543	9	Thick and thin blood films	CareStart (Access Bio, Inc., USA)	pf-HRP2/pan-pLDH	3	716	396	320	ND	ND	ND	ND
								ParaScreen (Zephyr Biomedicals, India)	pf-HRP2/pan-pLDH	3	719	383	336				
								ICT Combo (ICT Diagnostics, South Africa)	pf-HRP2/pan-aldolase	2	737	399	338				
3. Ref.^28^	Berzosa et al., 2018	Equatorial Guinea (2013)	Residents (1,741)	655	580	0	Thick and thin blood films	NADAL Malaria 4 species test (Test cassette) (Nal von Minden, Germany)	pf-HRP2/pan-pLDH	3	761	527	212	Semi-nested multiplex PCR (18S rRNA)	787	772	15
4. Ref.^19^	Bouyou et al., 2014	Gabon (2013)	Febrile patients (287)	94	93	1	Thick and thin blood films	SD BIOLINE Malaria Ag -Pf/Pan (Standard Diagnostics Inc., Republic of Korea)	pf-HRP2/pan-pLDH	3	103	102	1^a^	ND			
5. Ref.^48^	Chanie et al., 2011	Ethiopia (2009–2010)	Febrile patients (1,092)	226	224	2	Thick and thin blood films	CareStart Malaria Pf/Pv Combo test (Access Bio, Inc., USA)	pf-HRP2/Pv-pLDH	6	243	239	4^d^	ND			
6. Ref.^29^	Edwards et al., 2015	Cambodia (2013–2014)	Febrile patients (3,206)	ND	ND	ND	ND	SD BIOLINE Malaria Ag P.f/P.v (Standard Diagnostics Inc., Republic of Korea)	pf-HRP2/Pv-pLDH	6	103	101	2	Reverse Transcription PCR (18S rRNA)	174	154	20
7. Ref.^21^	Ehtesham et al., 2015	Iran (2012)	Malaria-positive (100)	100	98	2	Thick and thin blood films	First Response Malaria Antigen pLDH/HRP2 Combo (Premier Medical, India)	pf-HRP2/pan-pLDH	3	91	77	14	Nested PCR (18S rRNA)	100	88	12
								CareStart Malaria HRP-2/pLDH (Pf/pan) Combo (Access Bio, Inc., USA)	pf-HRP2/pan-pLDH	3	90	78	12				
								CareStart Malaria HRP2/pLDH(Pf/Pv) Combo (Access Bio, Inc., USA)	pf-HRP2/Pv-pLDH	6	94	87	7^d^				
8. Ref.^30^	Endeshaw et al., 2010	Ethiopia (2007)	Febrile patients (1997)	475	391	84	Thick and thin blood films	ParascreenPan/Pf (Zephyr Biomedicalsystems, India)	pf-HRP2/pan-pLDH	3	372	297	75^a^	ND			
9. Ref.^31^	Feleke et al., 2017	Ethiopia (2015–2016)	Febrile patients (320)	41	36	5	Thick and thin blood films	CareStart Malaria HRP2/pLDH (Pf/PAN) Combo (Access Bio, Inc., USA)	pf-HRP2/pan-pLDH	3	43	38	5^a^	ND			
10. Ref.^45^	Getnet et al., 2015	Ethiopia (2014)	Febrile patients (359)	ND	ND	ND	ND	CareStart Malaria HRP2/pLDH (Pf/PAN) Combo (Access Bio, Inc., USA)	pf-HRP2/pan-pLDH	3	80	66	14	Not reported	116	79	6
11. Ref.^32^	Haanshuus et al., 2016	India (2011–2012)	Febrile patients (1,564)	ND	ND	ND	ND	ParaHIT-Total Ver. 1.0 Device 55IC204-10 (Span Diagnostics Ltd, India)	pf-HRP2/pan-aldolase	2	75	46	27	Nested PCR (18S rRNA)	268	221	30
12. Ref.^33^	Imwong et al., 2015	Vietnam	Residents (2,177)	229	225	0	Thick and thin blood films	SD BIOLINE Malaria Ag P.f/Pan POCT (Standard Diagnostics Inc., Republic of Korea)	pf-HRP2/pan-pLDH	3	224	216	8	Quantitative real-time PCR (18S rRNA)	988	521	56
13. Ref.^34^	Iqbal et al., 2001	Kuwait (1997–1998)	Febrile patients (515)	163	151	12	Thick and thin blood films	OptiMAL (Pf-pLDH/pan-pLDH) (Biorad, France)	Pf-LDH/pan-pLDH	4	142	134	8^c^	ND			
14. Ref.^46^	Iqbal et al., 2002	Kuwait (1999–2002)	Febrile patients (750)	271	247	24	Thick and thin blood films	ICT Malaria Pf/Pv (ICT Diagnostics, Australia)	pf-HRP2/Pv-pLDH	6	178	166	12^d^	ND			
								OptiMAL (Biorad, France)	Pf/Pv -pLDH	5	230	212	18				
15. Ref.^35^	Jahan et al., 2019	Pakistan (2013)	Febrile patients (2,033)	359	320	39	Thick and thin blood films	First response Malaria Ag, pLDH/HRP2 Combo Card test kit (Premier Medical Corporation Ltd.)	pf-HRP2/pan-pLDH	3	266	22	39^a^	Nested PCR (18S rRNA)	95	95	0
16. Ref.^47^	Khorashad et al., 2014	Iran (2009–2010)	Febrile patients (178)	52	47	5	Thick and thin blood films	Malaria 102 (p.f/p.v) POCT kits (InTec Products Inc., China)	pf-HRP2/pan-pLDH	3	40	35	5^a^	ND			
17. Ref.^36^	Kim et al., 2008	Korea (2003–2007)	Malaria-positive (182) Healthy (100)	182	179	0	Thick and thin blood films	OptiMAL (Biorad, France)	Pf-LDH/pan-pLDH	4	174	171	3^c^	Conventional PCR (PvMSP-1, PfCSP-1)	ND	ND	3
								SD Malaria Antigen Pf/Pv (Standard Diagnostics Inc., Republic of Korea)	pf-HRP2/Pv-pLDH	6	172	169	3^d^				
18. Ref.^37^	Li et al., 2016	China (2011–2012)	Febrile patients (103)	ND	ND	ND	ND	Malaria Pv/Pf Test Device, Tycolpharm Co., Limited, UK)	pf-HRP2/Pv-pLDH	6	61	60	1	Nested PCR (18S rRNA)	69	66	3
19. Ref.^49^	Meena et al., 2009	India (2007)	Febrile patients (1,189)	71	69	2	Thick and thin blood films	FalciVax (Orchid Biomedical Laboratories, India)	pf-HRP2/Pv-pLDH	6	75	74	1^d^	ND			
20. Ref.^44^	Mehlotra et al., 2019	Madagascar (2015–2016)	Febrile patients (963)	452	452	0	Thick and thin blood films	SD BIOLINE Malaria Ag P.f/Pan (Standard Diagnostics Inc., Republic of Korea)	pf-HRP2/pan-pLDH	3	461	89	372	PCR/LDR-FMA	559	535	24
21. Ref.^38^	Moges et al., 2012	Ethiopia (2011)	Febrile patients (254)	114	98	6	Thick and thin blood films	CareStart Malaria HRP2/pLDH (Pf/pan) (Access Bio, Inc., USA)	pf-HRP2/pan-pLDH	3	100	74	26	ND			
22. Ref.^39^	Ranjan P. and Ghoshal U, 2016	India (2013–2015)	Febrile patients (561)	64	64	0	Thick and thin blood films	Not reported	Not reported	Not reported	92	89	3	Nested PCR (18S rRNA)	78	75	3
23. Ref.^40^	Ratnawati et al., 2008	Indonesia (2006)	Febrile patients (89)	78	56	22	Thick and thin blood films	Rapid One-Step Malaria test (Arista Biologicals Inc., USA)	pf-HRP2/pan-pLDH	3	72	50	22^a^	ND			
24. Ref.^41^	Richter et al., 2004	Germany (1999–2004)	Febrile patients (674)	69	65	4	Thick and thin blood films	The Now Malaria test (Binax, Inc., USA)	pf-HRP2/pan-aldolase	2	59	28	4^b^	ND			
25. Ref.^50^	Sharew et al., 2009	Ethiopia (2008)	Febrile patients (668)	314	304	10	Thick and thin blood films	CareStart Malaria Pf/Pv Combo test (Access Bio, Inc., USA)	pf-HRP2/Pv-pLDH	6	331	321	10^d^	ND			
	van den Broek et al., 2006	Colombia (2005)	Febrile patients (896)	140	133	7	Thick and thin blood films	Optimal-IT (Diamed AG, Switzerland)	Pf-LDH/pan-pLDH	4	134	128	7^c^	ND			
26. Ref.^51^								NOW Malaria ICT (Binax, USA)	pf-HRP2/pan-aldolase	2	134	126	8^b^	ND			
27. Ref.^42^	Woyessa et al., 2013	Ethiopia (2008–2010)	Febrile patients (2,394)	479	474	5	Thick and thin blood films	CareStart Malaria Pf/Pv combo test (Access Bio, Inc., USA)	pf-HRP2/Pv-pLDH	6	686	672	14^d^	ND			
28. Ref.^43^	Yan et al., 2013	Myanmar (2011)	Febrile patients (350)	98	87	11	Thick and thin blood films	Wondfo One Step Malaria HRP2/pLDH (P.f/Pan) Test	pf-HRP2/pan-pLDH	3	93	60	33	Nested PCR (18S rRNA)	113	92	21
								Malaria Pv/Pf test device (Tycolpharm Co., Limited, UK)	pf-HRP2/Pv-pLDH	6	90	82	8^d^				

18 studies in total yielded identical results with RDT and microscopy.

*ND* not determine.

^a^Six studies (4, 8, 9, 15, 16, and 23) yielded identical results with RDT type 3 and microscopy.

^b^Two studies (24 and 26) yielded identical results for RDT type 2 and microscopy.

^c^Three studies (13, 17, and 26) yielded identical results for RDT type 4 and microscopy.

^d^Nine studies (1, 5, 7, 14, 17, 19, 25, 27, and 28) yielded identical results for RDT type 6 and microscopy.

### WHO product testing of malaria RDTs

The WHO product testing of malaria RDTs began in 2008^52^. All companies manufacturing malaria RDTs under the ISO-13485 Quality System Standard were invited to submit up to three tests for evaluation^52^. The results of the WHO product testing of malaria RDTs are demonstrated in Table 2. RDTs from the eight studies^30,34,36,40,41,46,49,51^ were not subject to the WHO product testing program as these RDTs were developed and used before 2008, while the results of malaria RDTs from the four studies^28,37,43,47^ was not found on the WHO testing product.

**Table 2:** Characteristic of *Plasmodium* mixed infections.

No. (ref.)	Authors	Microscopy			RDTs						PCR results	
		Positivity	*Plasmodium* spp.	Blood collection methods	Manufacturers	Antigen	WHO product testing	False positive (%) *Plasmodium* spp. infection in clean-negative samples	Number of mixed infections	Types of mixed infections	Number of mixed infections	Types of mixed infections
1. Ref.^26^	Alam et al., 2011	3	*P. falciparum*/*P. vivax*	Venipuncture	Onsite Pf/Pv (CTK Biotech Inc, USA)	pf-HRP2/Pv-pLDH	Round 2	0	5	*P. falciparum*/*P. vivax*	8	*P. falciparum*/*P. vivax* (8)
					FalciVax Pf (Zephyr Biomedicals, India)	pf-HRP2/Pv-pLDH	Round 2	4.5	2	*P. falciparum*/*P. vivax*		
2. Ref.^27^	Ashton et al., 2010	9	*P. falciparum*/*P. vivax*	Finger prick	CareStart (Access Bio, Inc., USA)	pf-HRP2/pan-pLDH	Round 1	3.0	320	*P. falciparum*/*P. vivax*		
					ParaScreen (Zephyr Biomedicals, India)	pf-HRP2/pan-pLDH	Round 1	1.2	336	*P. falciparum*/*P. vivax*		
					ICT Combo (ICT Diagnostics, South Africa)	pf-HRP2/pan-aldolase	Round 1	0.6	338	*P. falciparum*/*P. vivax*		
3. Ref.^28^	Berzosa et al., 2018	0		Finger prick	NADAL Malaria 4 species test (Test cassette) (Nal von Minden, Germany)	pf-HRP2/pan-pLDH	Not found in WHO product testing records	Not found in WHO product testing records	212	Not specified	15	Not specified
4. Ref.^19^	Bouyou et al., 2014	1	*P. falciparum*/*P. malariae*	Venipuncture	SD BIOLINE Malaria Ag -Pf/Pan (Standard Diagnostics Inc., Republic of Korea)	pf-HRP2/pan-pLDH	Round 4	1.3	1	*P. falciparum*/*P. malariae*		
5. Ref.^48^	Chanie et al., 2011	2	*P. falciparum*/*P. vivax*	Finger prick	CareStart Malaria Pf/Pv Combo test (Access Bio, Inc., USA)	pf-HRP2/Pv-pLDH	Round 2	0	4	*P. falciparum*/*P. vivax*		
6. Ref.^29^	Edwards et al., 2015				SD BIOLINE Malaria Ag P.f/P.v (Standard Diagnostics Inc., Republic of Korea)	pf-HRP2/Pv-pLDH	Round 4	2.8	2	*P. falciparum*/*P. vivax*	20	*P. falciparum*/*P. vivax* (19), *P. vivax*/*P. malariae* (1)
7. Ref.^21^	Ehtesham et al., 2015	2	*P. falciparum*/*P. vivax*	Venipuncture	First Response Malaria Antigen pLDH/HRP2 Combo (Premier Medical, India)	pf-HRP2/pan-pLDH	Round 2	0	14	*P. falciparum*/*P. vivax*	12	*P. falciparum*/*P. vivax*
					CareStart Malaria HRP-2/pLDH (Pf/pan) Combo (Access Bio, Inc., USA)	pf-HRP2/pan-pLDH	Round 1	0	12	*P. falciparum*/*P. vivax*		
					CareStart Malaria HRP2/pLDH(Pf/Pv) Combo (Access Bio, Inc., USA)	pf-HRP2/Pv-pLDH	Round 2	0	7	*P. falciparum*/*P. vivax*		
8. Ref.^30^	Endeshaw et al., 2010	84	*P. falciparum*/*P. vivax*	Finger prick	ParascreenPan/Pf (Zephyr Biomedicalsystems, India)	pf-HRP2/pan-pLDH	Not assessed	Not assessed	75	*P. falciparum*/*P. vivax*		
9. Ref.^31^	Feleke et al., 2017	5	*P. falciparum*/*P. vivax*	Venipuncture	CareStart Malaria HRP2/pLDH (Pf/PAN) Combo (Access Bio, Inc., USA)	pf-HRP2/pan-pLDH	Round 5	0.4	5	*P. falciparum*/*P. vivax*		
10. Ref.^45^	Getnet et al., 2015				CareStart Malaria HRP2/pLDH (Pf/PAN) Combo (Access Bio, Inc., USA)	pf-HRP2/pan-pLDH	Round 5	0.4	14	*P. falciparum*/*P. vivax*	6	*P. falciparum*/*P. vivax* (5), *P. vivax*/*P. malariae* (1),
11. Ref.^32^	Haanshuus et al., 2016				ParaHIT-Total Ver. 1.0 Device 55IC204-10 (Span Diagnostics Ltd, India)	pf-HRP2/pan-aldolase	Round 4	0	27	*P. falciparum*/Pan	30	*P. falciparum*/*P. vivax* (27), *P. falciparum*/*P. malariae* (2), *P. vivax*/*P. malariae* (1),
12. Ref.^33^	Imwong et al., 2015	0		Venipuncture	SD BIOLINE Malaria Ag P.f/Pan POCT (Standard Diagnostics Inc., Republic of Korea)	pf-HRP2/pan-pLDH	Round 5	0	8	*P. falciparum*/*P. vivax*	56	*P. falciparum*/*P. vivax* (56)
13. Ref.^34^	Iqbal et al., 2001	12	*P. falciparum*/*P. vivax*	Not specified	OptiMAL (Pf-pLDH/pan-pLDH) (Biorad, France)	Pf-LDH/pan-pLDH	Not assessed	Not assessed	8	*P. falciparum*/*P. vivax*		
14. Ref.^46^	Iqbal et al., 2002	24	*P. falciparum*/*P. vivax*	Finger prick	ICT Malaria Pf/Pv (ICT Diagnostics, Australia)	pf-HRP2/Pv-pLDH	Not assessed	Not assessed	12	*P. falciparum*/*P. vivax*		
					OptiMAL (Biorad, France)	Pf/Pv -pLDH	Not assessed	Not assessed	18	*P. falciparum*/*P. vivax*		
15. Ref.^35^	Jahan et al., 2019	39	*P. falciparum*/*P. vivax*	Finger prick	First response Malaria Ag, pLDH/HRP2 Combo Card test kit (Premier Medical Corporation Ltd.)	pf-HRP2/pan-pLDH	Round 5	0	39	*P. falciparum*/*P. vivax*	0	
16. Ref.^47^	Khorashad et al., 2014	5	*P. falciparum*/*P. vivax*	Finger prick	Malaria 102 (p.f/p.v) POCT kits (InTec Products Inc., China)	pf-HRP2/pan-pLDH	Not found in WHO product testing records	Not found in WHO product testing records	5	*P. falciparum*/*P. vivax*		
17. Ref.^36^	Kim et al., 2008	0		Venipuncture	OptiMAL (Biorad, France)	Pf-LDH/pan-pLDH	Not assessed	Not assessed	3	*P. falciparum*/*P. vivax*	3	*P. falciparum*/*P. vivax*
					SD Malaria Antigen Pf/Pv (Standard Diagnostics Inc., Republic of Korea)	pf-HRP2/Pv-pLDH	Not assessed	Not assessed	3	*P. falciparum*/*P. vivax*		
18. Ref.^37^	Li et al., 2016				Malaria Pv/Pf Test Device, Tycolpharm Co., Limited, UK)	pf-HRP2/Pv-pLDH	Not found in WHO product testing records	Not found in WHO product testing records	1	*P. falciparum*/*P. vivax*	3	*P. falciparum*/*P. vivax*
19. Ref.^49^	Meena et al., 2009	2	*P. falciparum*/*P. vivax*	Finger prick	FalciVax (Orchid Biomedical Laboratories, India)	pf-HRP2/Pv-pLDH	Not assessed	Not assessed	1	*P. falciparum*/*P. vivax*		
20. Ref.^44^	Mehlotra et al., 2019	0		Finger prick	SD BIOLINE Malaria Ag P.f/Pan (Standard Diagnostics Inc., Republic of Korea)	pf-HRP2/pan-pLDH	Round 5	0	372	*P. falciparum*/Pan	24	*P. falciparum*/*P. vivax* (13), *P. falciparum*/*P. malariae* (5), *P. falciparum*/*P. ovale* (1), *P. malariae*/*P. ovale* (2), *P. falciparum*/*P. malariae*/*P. ovale* (3),
21. Ref.^38^	Moges et al., 2012	6	*P. falciparum*/*P. vivax*	Finger prick	CareStart Malaria HRP2/pLDH (Pf/pan) (Access Bio, Inc., USA)	pf-HRP2/pan-pLDH	Round 1	3.0	26	*P. falciparum*/Pan		
22. Ref.^39^	Ranjan P. and Ghoshal U, 2016	0		Venipuncture	Not reported	Not reported	–	–	3	*P. falciparum*/*P. vivax*	3	*P. falciparum*/*P. vivax*
23. Ref.^40^	Ratnawati et al., 2008	22	*P. falciparum*/*P. vivax*	Not specified	Rapid One-Step Malaria test (Arista Biologicals Inc., USA)	pf-HRP2/pan-pLDH	Not assessed	Not assessed	22	*P. falciparum*/*P. vivax*		
24. Ref.^41^	Richter et al., 2004	4	*P. falciparum*/*P. ovale* (3)*, P. falciparum*/*P. malariae* (1)	Not specified	The Now Malaria test (Binax, Inc., USA)	pf-HRP2/pan-aldolase	Not assessed	Not assessed	4	*P. falciparum*/*P. ovale* (3)*, P. falciparum*/*P. malariae* (1)		
25. Ref.^50^	Sharew et al., 2009	10	*P. falciparum*/*P. vivax*	Not specified	CareStart Malaria Pf/Pv Combo test (Access Bio, Inc., USA)	pf-HRP2/Pv-pLDH	Round 2	0.5	10	*P. falciparum*/*P. vivax*		
26. Ref.^51^	van den Broek et al., 2006	7	*P. falciparum*/*P. vivax*	Finger prick	Optimal-IT (Diamed AG, Switzerland)	Pf-LDH/pan-pLDH	Not assessed	Not assessed	7	*P. falciparum*/Pan		
					NOW Malaria ICT (Binax, USA)	pf-HRP2/pan-aldolase	Not assessed	Not assessed	8	*P. falciparum*/Pan		
27. Ref.^42^	Woyessa et al., 2013	5	*P. falciparum*/*P. vivax*	Not specified	CareStart Malaria Pf/Pv combo test (Access Bio, Inc., USA)	pf-HRP2/Pv-pLDH	Round 4	0	14	*P. falciparum*/*P. vivax*		
28. Ref.^43^	Yan et al., 2013	11	*P. falciparum*/*P. vivax*	Finger prick	Wondfo One Step Malaria HRP2/pLDH (P.f/Pan) Test	pf-HRP2/pan-pLDH	Round 1	0	33	*P. falciparum*/Pan	21	*P. falciparum*/*P. vivax*
					Malaria Pv/Pf test device (Tycolpharm Co., Limited, UK)	pf-HRP2/Pv-pLDH	Not found in WHO product testing records	Not found in WHO product testing records	8	*P. falciparum*/*P. vivax*		

### Methodological quality of the included studies

**Figure 2:**
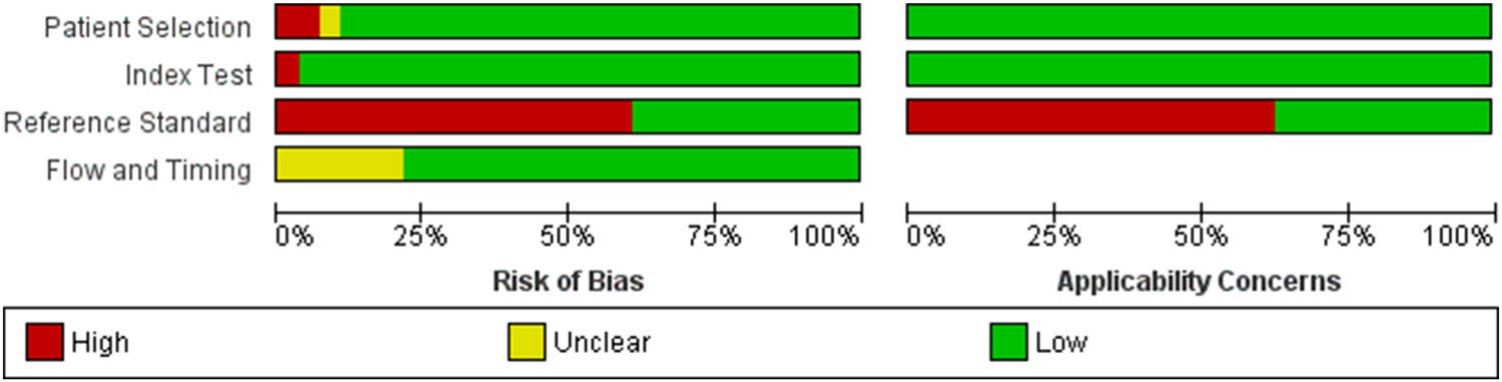
Methodological quality of the included studies.

The methodology and reporting of the selected studies varied highly (Fig. 2; Supplementary Fig. 1). All 28 included studies had cross-sectional designs. Most of the included studies (25/28, 89.3%) used a consecutive or random sample of patients. Two studies did not enrol a consecutive or random sample of patients^21,36^. In another study^19^, the sampling method for participants enrolled was unclear. Microscopic examination was used as the reference standard in 24 studies. PCR was used as a reference standard in 12 studies. The sensitivity and specificity of RDTs to detect *Plasmodium* mixed infections could not be calculated due to the inadequate data of the included studies to retrieve full 2 × 2 tables.

### Discordance between RDTs and microscopy

**Figure 3:**
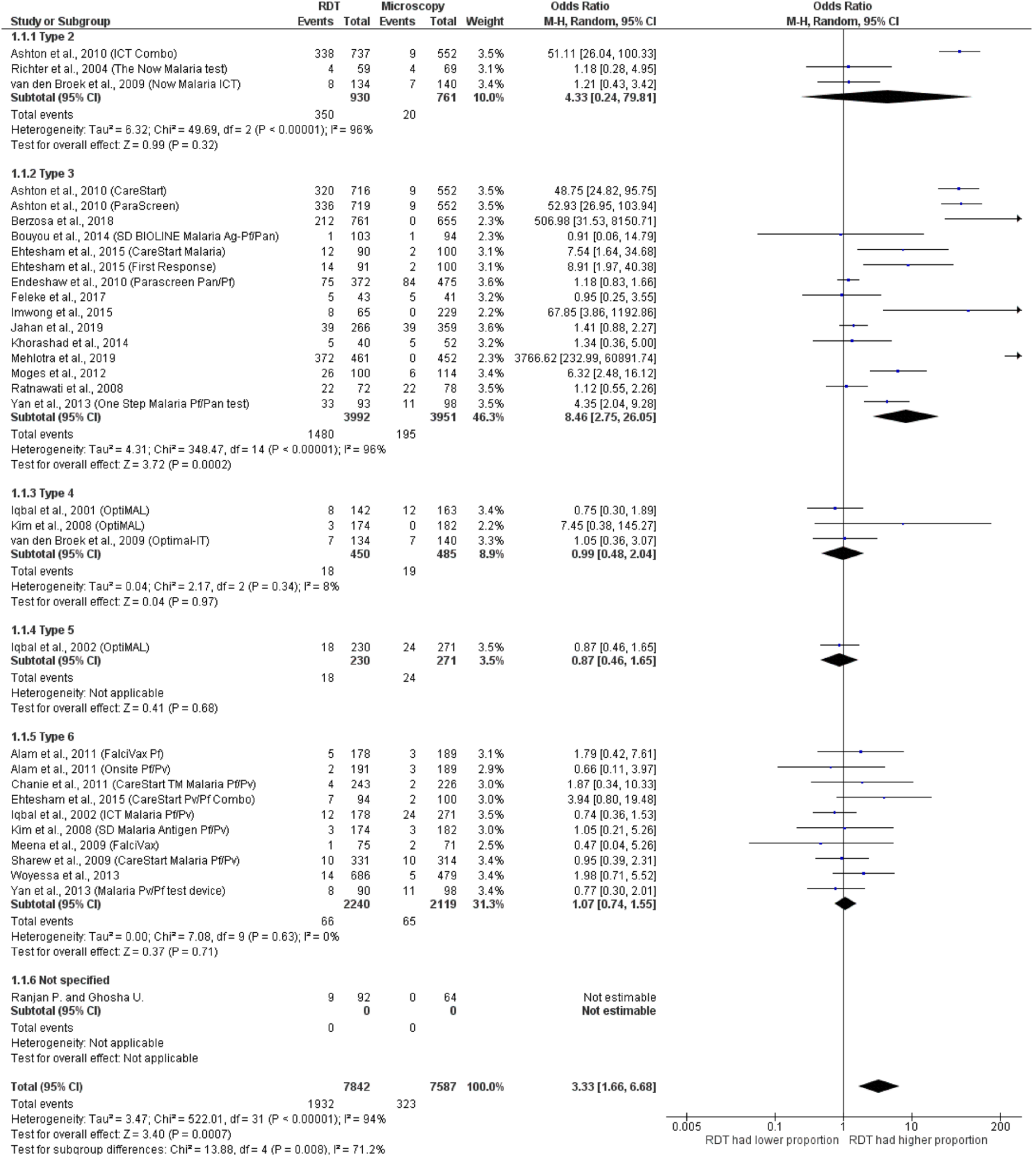
Discordance between RDTs and microscopy.

All 24 studies reporting on the performance of RDTs for detecting mixed infections compared to microscopy were explicitly designed for this purpose^19,21,26–28,30,31,33–36,38–44,46–51^ (Fig. 3). In total, six different RDT types including RDT types 2, 3, 4, 5 and 6 were included in the analysis. One study did not report the type of RDT used in their study^39^. Six studies used more than one RDT type/brand in their studies^21,26,27,35,36,43^. Four studies^28,33,36,44^ reported mixed infections by RDT, but no mixed infections were reported by microscopy. The results of an individual study demonstrated that 18 studies in total^19,21,26,30,31,34–36,40–43,46–51^ had identical results for RDT and microscopy. Six studies^19,30,31,35,40,47^ gave identical results for RDT type 3 and microscopy. Two studies^41,51^ gave identical results for RDT type 2 and microscopy. Three studies^34,36,51^ gave identical results for RDT type 4 and microscopy. Nine studies^21,26,36,42,43,46,48–50^ gave identical results for RDT type 6 and microscopy. The summary estimate of ORs between type 2 RDTs and microscopy to detect mixed infections ranged from 1.18 to 51.1. Based on the analysis of three included studies, the summary estimate of ORs between type 2 RDTs and microscopy was 4.33 (95% CI 0.24–79.8, p = 0.32, I^2^ = 96%). The summary estimate of ORs between type 3 RDTs and microscopy to detect mixed infections based on the analysis of 13 included studies was 8.46 (95% CI 2.75–26.1, p = 0.0002, I^2^ = 96%). When the four studies^28,33,39,44^ that reported mixed infections detected by RDT but did not reported mixed infections by microscopy were ignored in the meta-analysis of type 3 RDTs and microscopy, the summary estimate of ORs between type 3 RDTs and microscopy to detect mixed infections based on the analysis of 13 included studies was 4.02 (95% CI 1.46–11.12, p = 0.007, I^2^ = 95%) (Supplementary file 1). The summary estimate of ORs between type 4 RDTs and microscopy to detect mixed infections based on the analysis of three included studies, was 0.99 (95% CI 0.48–2.04, p = 0.97, I^2^ = 8%). The summary estimate of ORs between type 5 RDTs and microscopy to detect mixed infections based on the analysis of one included study was 0.87 (95% CI 0.46–1.65). The summary estimate of ORs between type 6 RDTs and microscopy to detect mixed infections based on the analysis of nine included studies was 1.07 (95% CI 0.74–1.55, p = 0.71, I^2^ = 0%). Overall, the significant summary estimate of ORs between all types of RDTs and microscopy to detect mixed infections was found (OR = 3.33, 95% CI 1.66–6.68, p = 0.009, I^2^ = 94%).

**Figure 4:**
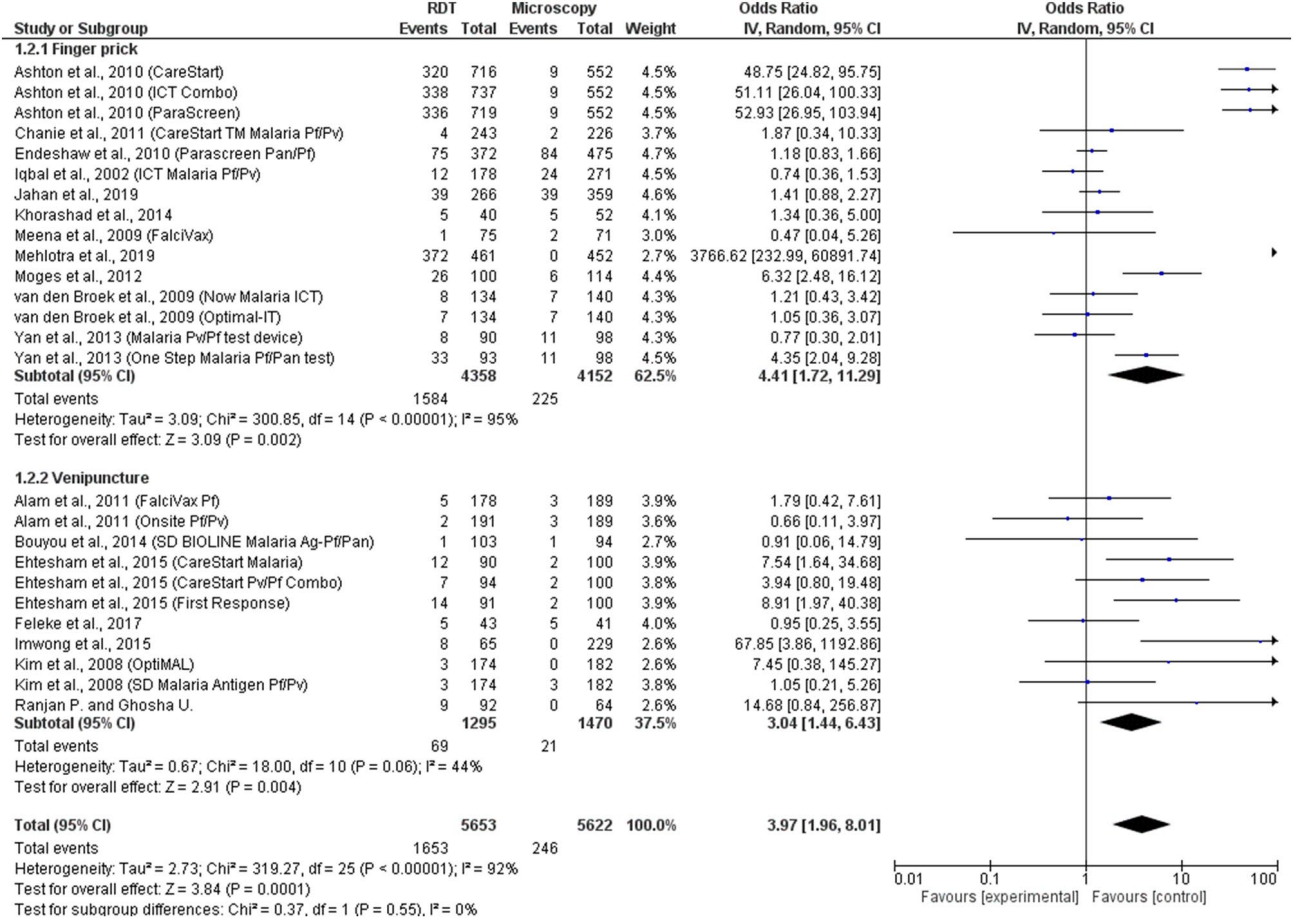
The subgroup analysis of blood collection methods for microscopy.

The subgroup analysis of blood collection methods for microscopy was performed using 18 included studies. The results demonstrated that no subgroup difference (p = 0.55) was found among studies using blood from the finger prick method and those using blood from venipuncture. The summary estimate of ORs between all types of RDTs compared to those performing microscopy using blood from the finger prick method to detect mixed infections was significantly different among 11 studies (OR = 4.41, 95% CI 1.72–11.29, p = 0.002). The summary estimate of ORs between RDTs and microscopy using blood from the venipuncture method was significantly different among seven studies (OR = 3.04, 95% CI 1.44–6.43, p = 0.004) (Fig. 4).

### Discordance between RDTs and PCR

**Figure 5:**
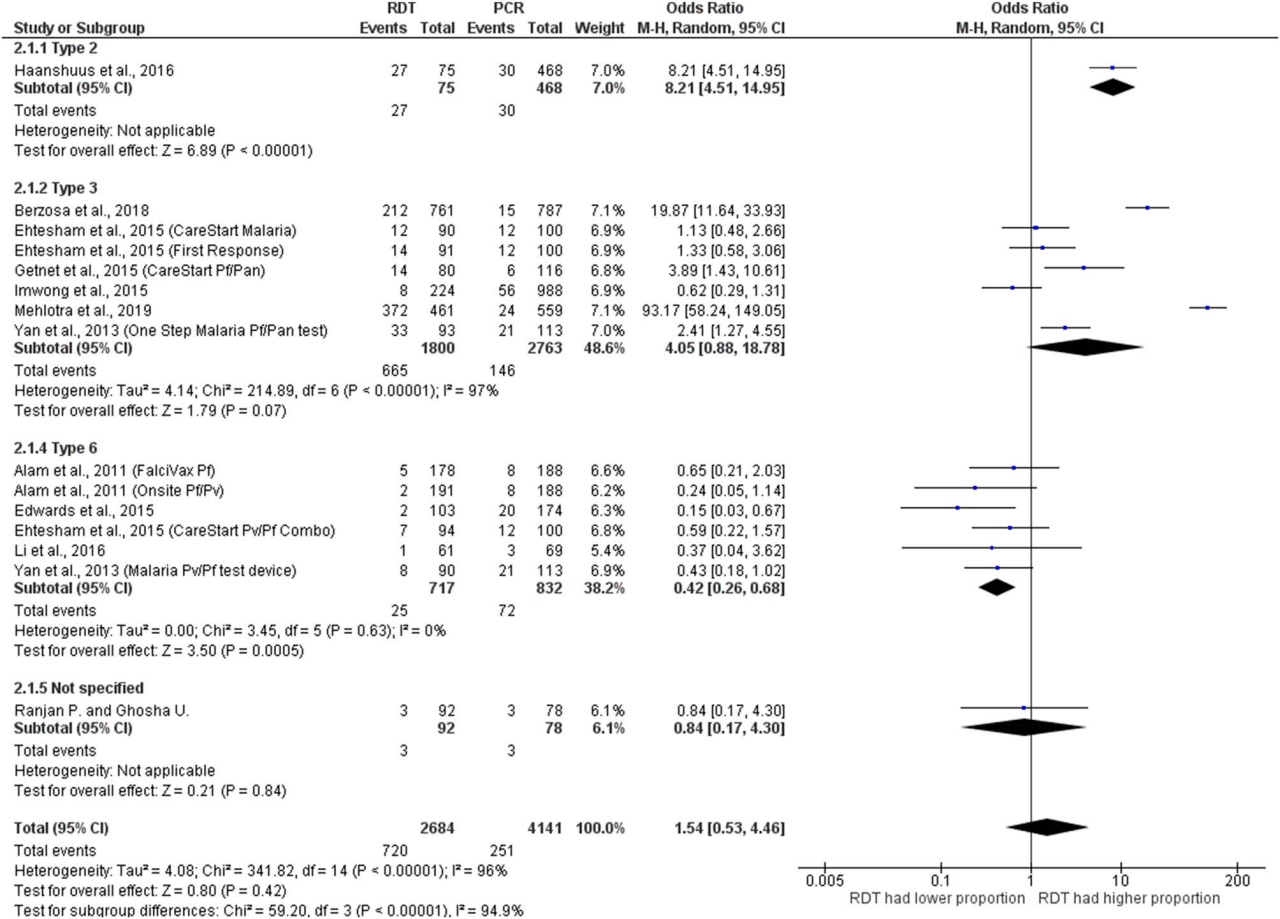
Discordance between RDTs and PCR.

**Figure 6:**
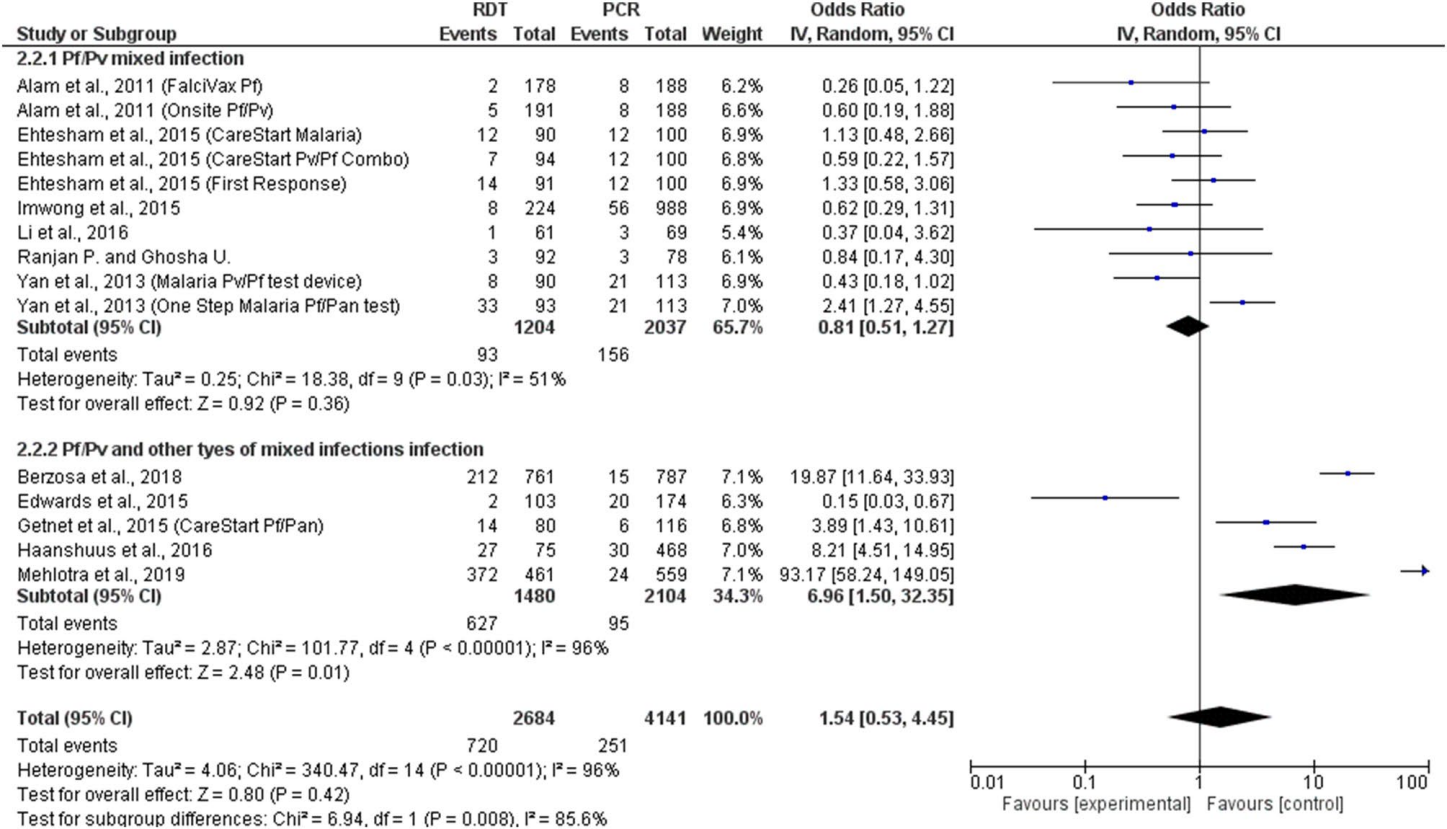
The subgroup analysis of detection of Plasmodium mixed-species infections between RDT and PCR.

Overall, 12 studies reported on mixed infections detected by both RDTs and PCR^21,26–29,32,33,37,39,43–45^, as shown in Fig. 5. Among 12 studies, three different types of RDTs were reported including RDT types 2, 3, and 6. The summary estimate of ORs between type 2 RDTs and PCR to detect mixed infections was 8.21 (95% CI 4.51–15.0). The summary estimate of ORs between type 3 RDTs and PCR based on the analysis of six included studies to detect mixed infections was 4.05 (95% CI 0.73–7.84, p = 0.07, I^2^ = 97%). The summary estimate of ORs between type 6 RDTs and PCR based on the analysis of five included studies to detect mixed infections was 0.42 (95% CI 0.26–0.68, p = 0.0005, I^2^ = 0%). Another study with no description on the type of RDT showed that the summary estimate of ORs between RDTs and PCR to detect mixed infections was 0.84 (95% CI 0.17–4.3). Overall, the summary estimate of ORs between all types of RDTs and PCR to detect mixed infections was 1.17 (95% CI 1.54–0.53, p = 0.42, I^2^ = 96%). The subgroup analysis of detection of *Plasmodium* mixed-species infections between RDT and PCR found that the summary estimate of ORs between RDT and PCR was comparable in *P. falciparum* mixed infections with *P. vivax* (OR= 0.81, 95% CI 0.51–1.27, p = 0.36, I^2^ = 51%) and in *P. falciparum*/*P. vivax* and *P. falciparum* mixed infections with other *Plasmodium* spp. (OR: 6.96, 95% CI 1.50–32.4, p = 0.01, I^2^: 99%) (Fig. 6).

### Publication bias

**Figure 7:**
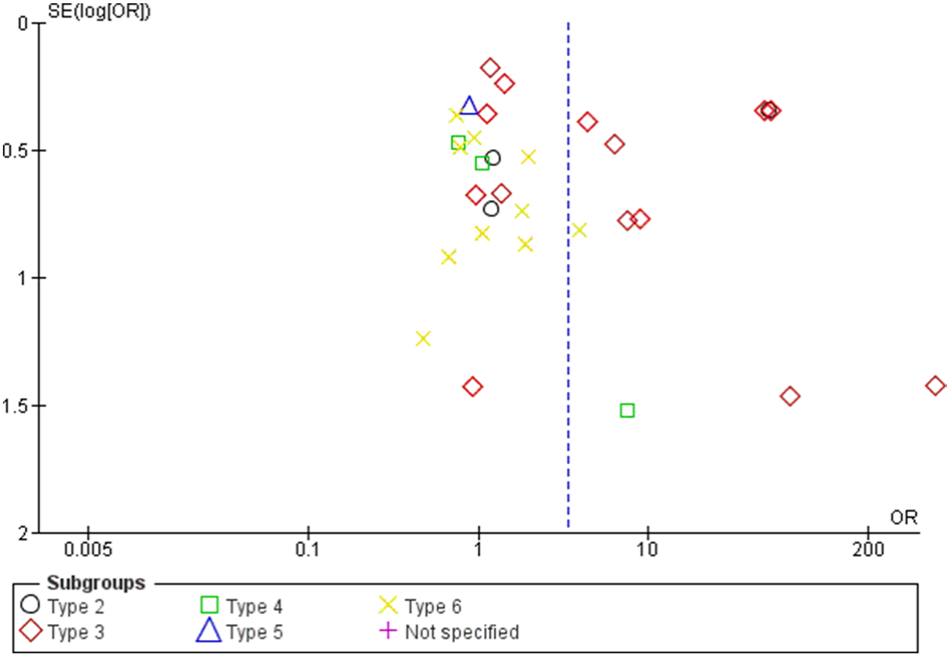
The funnel plot.

Visual inspection of the funnel plots demonstrated no publications bias found because there was a symmetrical distribution of the included studies (geometric shapes) in the graph between the OR and SE (logOR) (Fig. 7). The publication bias was further assessed with Egger's test. Egger's test showed no publication bias due to the small-study effects found (p-value = 0.166) (Table S2). Therefore, the summary estimates of ORs in the present meta-analysis were not confounded by publication bias of the included studies.

## Discussion

This is the first study to summarize the available data on the discrepancy between RDTs and two gold/reference standards for the detection of malaria mixed infections. The summary ORs of discrepancies of RDT types 2, 3, 4, 5, and 6 in detecting malaria mixed infections compared to microscopy were 4.33, 8.46, 0.99, 0.87, and 1.07, respectively. Even though the overall summary estimate of ORs was significantly observed, subgroup analysis of RDT types demonstrated that only RDT type 3 could detect a significantly higher proportion of *Plasmodium* mixed infections than the microscopic method. Among the 8 studies conducted in Ethiopia^27,30,31,38,42,45,48,50^, only a study by Ashton et al.^27^ revealed a considerable difference in the proportion of mixed infections detected by RDT types 2 and 3 compared with microscopy. From 297 blood samples of *P. falciparum* mono-infection confirmed by the microscopy, 213 (213/297: 71.7%), 224 (224/297: 75.4%), and 223 (223/297: 75.1%) samples were interpreted as mixed infections by CareStart (AccessBio, USA), ICT Combo (ICT Diagnostics, South Africa), and ParaScreen (Zephyr Biomedicals, India), respectively. The remaining studies conducted in Ethiopia had identical numbers of mixed infections in 2 studies^31,50^ and high numbers of mixed infections in three studies^38,42,48^,another study conducted in Ethiopia demonstrated more mixed infections detected by microscopy (84 cases) than by RDT (75 cases)^30^. Another important difference in the proportion of mixed infections detected by RDT type 3 compared with microscopy was also demonstrated in the study conducted in Madagascar by Mehlotra et al., 2019 during 2015–2016 because 84.6% of blood samples with confirmed *P. falciparum* mono-infections by microscopy and by LDR-FMA analysis were positive for both the Pf-HRP2 and pan-pLDH test bands^44^. In addition, an important difference in the proportion of mixed infections detected by RDT type 3 compared with microscopy was also demonstrated in the study conducted in Madagascar by Berzosa et al. because 0.87% of blood samples with confirmed *P. falciparum* mixed infections by PCR were false positive for *Plasmodium* mixed infections by RDT type 3 (212 cases, 12.3%)^28^.

The high proportion of mixed infections detected by RDT types 2 and 3 compared with microscopy reported in the included studies by Ashton et al., Mehlotra et al., and by Berzosa et al. may be due to the consistent false positive Pan-pLDH test lines among *P. falciparum* samples at high parasite densities, as reported in RDTs targeting Pv-pLDH^53^. A high parasite density of *P. falciparum* can induce positivity of the pLDH band on RDTs, giving false positives of non-falciparum species^28^. The false positive on Pan-pLDH test lines among *P. falciparum* samples at high parasite densities may be possible to use as the detection limit of the SD Bioline Malaria Ag P.f/Pan RDT used in the study by Mehlotra et al. because the mean parasitaemia level in samples that were positive for both the PfHRP2 and pan-pLDH test bands was significantly higher than that in those that were positive only for the PfHRP2 band^44^. In addition, the included study by Ashton et al., 2010, demonstrated the false-positive results in Pan-pLDH test lines of *P. falciparum* (38%) and *P. vivax* samples which might cause by high parasite densities (> 5,000 parasites/µl)^27^. Therefore, high *P. falciparum* or *P. vivax* parasitaemia could lead to incorrect interpretation of RDTs, particularly interpretation of mixed infections. The discordance between RDT types 2 or 3 and microscopy can be explained because RDT type 3 is specific to Pf-HRP2 and pan-pLDH and RDT type 2 is specific to pan-aldolase and thus cannot distinguish between a *P. falciparum* infection and a mixed infection when both test lines are observed. Other possible causes of discrepancy were false positive results from patients who had received any anti-malarial treatment in the previous four weeks as reported by the authors, parasitized erythrocytes cytoadhered to the microvasculature that were not seen in the peripheral circulation or on blood films although antigen continued to be released yielding RDT positivity^54^, or a low parasite density of the mixed infection that was too low to be seen by the microscopists but with sufficient parasite antigen to yield RDT positivity^55^.

The meta-analysis of RDTs and microscopy had no significant discrepancy among RDTs type 2, 4, and 6. In this analysis, the summary results of RDT type 5 performed by Iqbal et al.^46^ and RDTs performed by Ranjan and Ghoshal^39^ could not be interpreted because there were a small number of studies for subgroup analysis. Overall, the evidence was strong for RDT types 3 and 6 mainly because a large number of studies were available for inclusion. However, the summary estimate of RDT type 3 demonstrated high heterogeneity among the included studies (I^2^ = 96%) when compared to those of RDT type 6 (I^2^ = 0%). In this study, more than half of the studies (n = 18) relied solely on microscopy as the gold/reference standard for *Plasmodium* species identification. Therefore, the discordant results between RDTs and microscopy demonstrated in the present study might be due to the imperfect nature of the gold/reference standard because mixed infections with *P. falciparum* could be missed by microscopy. Because of these results, RDT types 2 and 3 could rectify the diagnosis of *P. falciparum* in mixed-species infections that might be missed by the microscopy method. These results supported that the selection of the most appropriate RDTs relative to malaria epidemiology and are very crucial to differentiated mixed infections because the identification of *Plasmodium* mixed-species infections would facilitate appropriate treatment with artemisinin-based combination therapies (ACTs), which could eliminate any mixed infection even if mixed infections were not detected by the gold/reference standard, the microscopy method^56^.

Recently, the sensitivity and specificity for the detection and identification of malarial parasites have been improved using the Nested-PCR method, which amplifies the *18s rRNA* gene^57^. It has been proven to be more sensitive and accurate than routine diagnostic microscopy and provides the advantage of a higher proportion of detection in cases of mixed-species infections^57^. In the present study, 12 included studies used PCR as a reference standard for *Plasmodium* species identifications. The discrepancy between RDT type 3 and PCR (OR = 4.05) appeared to be heavily influenced by the included studies by Berzosa et al. and by Mehlotra et al. in which the individual ORs were extremely high (19.9 and 93.2, respectively). This affirms that when compared with using PCR as the gold/reference standard, the high discrepancy between RDT type 3 targeting Pf-HRP2 and pan-pLDH leads to incorrect interpretation of mixed infections by RDTs, as we discussed earlier in the discrepancy of RDT type 3 and microscopy. The false positive results of RDTs when detecting mixed infections may be associated with decreased age because of the high prevalence of malaria in children, particularly children under 5 years of age, who are likely to develop severe malaria with high parasitaemia^58,59^. The present meta-analysis demonstrated that the significant discordance between RDTs and PCR was found in studies using RDT type 6, which detects the pf-HRP2/Pv-pLDH antigen of malaria parasites. RDT type 6 could detect a lower proportion of *Plasmodium* mixed infections than the PCR reference method. This finding was similar to three previous studies^21,60,61^. Therefore, the lower proportion of *Plasmodium* mixed infections detected by RDT type 6 than by PCR demonstrated in the present study might be due to the lower sensitivity and specificity of RDTs than of PCR methods. In practice, PCR methods have a higher sensitivity (approximately 0.0001 parasites/µL) than RDT (approximately 100 parasites/µL) and microscopy (approximately 50–500 parasites/µL)^8^, which allows for the detection of *Plasmodium* mixed infections at a low parasite density, which are routinely missed in microscopy^62^. The subgroup analysis of *Plasmodium* mixed-species infections as reported by the 6 included studies demonstrated that a comparable proportion detected *P. falciparum* mixed infections with *P. vivax* between RDTs and PCR, while there was a significant difference in the proportion that detected *P. falciparum*/*P. vivax* and *P. falciparum* mixed infections with other *Plasmodium* species. This subgroup analysis suggested that RDTs had identical results with PCR in detecting *P. falciparum* and *P. vivax* mixed infections. In contrast, RDTs had discordant results with PCR in detecting *P. falciparum* mixed infections with other *Plasmodium* species. Nevertheless, these results should be further confirmed by full experimental studies.

The present study had limitations. First, RDTs targeting HRP-2 and pan-pLDH or RDTs targeting HRP-2 and pan-aldolase are likely to be positive in *P. falciparum* mono-infections or mixed-species infections. Regarding this limitation of the RDTs in the included studies, the summary estimates of ORs between RDT types 2 and 3 and microscopy need to be carefully interpreted. Second, the overall evidence of the analysis between RDTs and PCR was weak, mainly because few studies were available for inclusion. Second, the lower sensitivity and specificity of RDTs than those of PCR was due to the limits of detection. The WHO has suggested that the clinical sensitivity of RDTs is highly dependent on conditions including the level of parasite density and the subset of any population, such as young children or pregnant women; thus, the interpretation of RDTs must be carefully interpreted^63^. Third, the sensitivity and specificity of RDTs compared to the gold standard could not be calculated due to data on individual patient were lacking and the data on whether patients who gave positive results for RDT were the same patients who gave positive results for the gold/reference standard or not, as most of the included studies report the number of positive separately between RDTs and microscopy/PCR. Fourth, some eligible studies might have been missed through the search strategy. However, the additional search of reference lists of the included studies and searches of other sources such as Google search and Google Scholar, and performing extensive searching of reference lists and searching other sources with broad search terms, helped to reduce this limitation by further increasing the number of included studies. Fifth, the study aimed to clarify what proportion of *Plasmodium* mixed-infections could not be confirmed by a positive RDT result, and the proportion of *Plasmodium* mixed infections were often not the primary target of studies, which led to a low number of studies that were focused on mixed infections. In light of these, although the current data are still suggestive of high discrepancies of RDT type 3 for detecting *Plasmodium* mixed infections in comparison to microscopy and of RDT type 6 for detecting *Plasmodium* mixed infections in comparison to PCR methods, they provided a critical advantage on malaria treatment in resource-limited settings in which the results of microscopy could not be obtained. Further studies focused on the diagnosis of *Plasmodium* mixed-species infections by RDTs are needed to provide a better understanding of the performance of RDTs, guide the development of an improved diagnostic test for *Plasmodium* mixed infections, and facilitate the appropriate treatment of patients with ACTs. This will help with the elimination of malaria in endemic and non-endemic areas where laboratory capacity is limited.

## Conclusion

In conclusion, the present study suggested that malaria RDTs showed some discordant results with microscopy and PCR. The selection interpretation of RDTs can facilitate a better diagnosis of *Plasmodium* mixed-species infections and appropriate treatment of malaria patients in endemic and non-endemic settings.

### Consent for publication

All authors have read the manuscript and consent to its publication.

## Supplementary information

Supplementary file 1

Supplementary Table S1.

Supplementary Table S2.

## Data Availability

The datasets used during the current study are available without restriction and demonstrated in the present manuscript and additional files.

## Abbreviations

RDTs: Rapid diagnostic tests
PCR: Polymerase chain reaction
CI: Confidence interval
OR: Odds ratio
HRP2: Histidine-rich protein 2
LDH: Lactate dehydrogenase
QUADAS: Quality assessment of diagnostic accuracy studies

## References

[1] WHO. World malaria report 2019. https://www.who.int/publications-detail/world-malaria-report-2019 (2019).

[2] Singh B, Daneshvar C (2013). Human infections and detection of *Plasmodium knowlesi*. Clin. Microbiol. Rev..

[3] Roper C (1996). Detection of very low level *Plasmodium falciparum* infections using the nested polymerase chain reaction and a reassessment of the epidemiology of unstable malaria in Sudan. Am. J. Trop. Med. Hyg..

[4] Looareesuwan S, White NJ, Chittamas S, Bunnag D, Harinasuta T (1987). High rate of *Plasmodium vivax* relapse following treatment of falciparum malaria in Thailand. Lancet.

[5] McKenzie FE, Bossert WH (1999). Multispecies *Plasmodium* infections of humans. J. Parasitol..

[6] Lee GC (2011). Development and evaluation of a rapid diagnostic test for *Plasmodium falciparum*, *P vivax*, and mixed-species malaria antigens. Am. J. Trop. Med. Hyg.

[7] Bell D, Wongsrichanalai C, Barnwell JW (2006). Ensuring quality and access for malaria diagnosis: how can it be achieved?. Nat. Rev. Microbiol..

[8] Moody A (2002). Rapid diagnostic tests for malaria parasites. Clin. Microbiol. Rev..

[9] Rock EP (1987). Comparative analysis of the *Plasmodium falciparum* histidine-rich proteins HRP-I, HRP-II and HRP-III in malaria parasites of diverse origin. Parasitology.

[10] Makler MT, Piper RC, Milhous WK (1998). Lactate dehydrogenase and the diagnosis of malaria. Parasitol. Today.

[11] Kanwugu ON (2019). Prevalence of asymptomatic malaria among children in the Tamale Metropolis: how does the PfHRP2 CareStart RDT perform against microscopy?. J. Trop. Med..

[12] Mweu MM, Wambua J, Njuga F, Bejon P, Mwanga D (2019). Bayesian evaluation of the performance of three diagnostic tests for *Plasmodium falciparum* infection in a low-transmission setting in Kilifi County Kenya. Wellcome Open. Res..

[13] Niyibizi JB, Gatera EK (2020). Diagnostic performance between histidine-rich protein 2 (HRP-2), a rapid malaria diagnostic test and microscopic-based staining techniques for diagnosis of malaria. J. Trop. Med..

[14] Abdalla ZA (2019). The diagnostic performance of rapid diagnostic tests and microscopy for malaria diagnosis in eastern Sudan using a nested polymerase chain reaction assay as a reference standard. Trans. R Soc. Trop. Med. Hyg..

[15] Coldiron ME (2019). Clinical diagnostic evaluation of HRP2 and pLDH-based rapid diagnostic tests for malaria in an area receiving seasonal malaria chemoprevention in Niger. Malar. J..

[16] Leski TA (2020). Use of real-time multiplex PCR, malaria rapid diagnostic test and microscopy to investigate the prevalence of Plasmodium species among febrile hospital patients in Sierra Leone. Malar. J..

[17] Dzakah EE (2014). Comparative performance of aldolase and lactate dehydrogenase rapid diagnostic tests in *Plasmodium vivax* detection. Malar. J..

[18] Bwire GM (2019). Diagnostic performance of CareStart malaria HRP2/pLDH test in comparison with standard microscopy for detection of uncomplicated malaria infection among symptomatic patients Eastern Coast of Tanzania. Malar. J..

[19] Bouyou-Akotet MK, Nkare CA, Mbouoronde OC, Mawili-Mboumba DP (2014). Performances of SD Bioline Malaria Ag-P.F/Pan RDT for the diagnosis of malaria in febrile patients living in Gabon, central Africa. Malar Chemoth Cont Elimin..

[20] Barber BE (2013). Evaluation of the sensitivity of a pLDH-based and an aldolase-based rapid diagnostic test for diagnosis of uncomplicated and severe malaria caused by PCR-confirmed *Plasmodium knowlesi*, *Plasmodium falciparum*, and *Plasmodium vivax*. J. Clin. Microbiol..

[21] Ehtesham R, Fazaeli A, Raeisi A, Keshavarz H, Heidari A (2015). Detection of mixed-species infections of *Plasmodium falciparum* and *Plasmodium vivax* by nested PCR and rapid diagnostic tests in southeastern Iran. Am. J. Trop. Med. Hyg..

[22] Whiting PF (2011). QUADAS-2: a revised tool for the quality assessment of diagnostic accuracy studies. Ann. Intern. Med..

[23] Rothstein HR, S. A., Borenstein M. *Publication bias in meta-analysis*. (Wiley, 2005).

[24] Lane DM (1978). Estimating effect size: Bias resulting from the significance criterion in editorial decisions. Br. J. Math. Stat. Psychol..

[25] Cochrane. Cochrane handbook for systematic reviews of interventions, https://training.cochrane.org/handbook/archive/v5.1/ (2011).

[26] Alam MS (2011). Real-time PCR assay and rapid diagnostic tests for the diagnosis of clinically suspected malaria patients in Bangladesh. Malar. J..

[27] Ashton RA (2010). Performance of three multi-species rapid diagnostic tests for diagnosis of *Plasmodium falciparum* and *Plasmodium vivax* malaria in Oromia Regional State Ethiopia. Malar. J..

[28] Berzosa P (2018). Comparison of three diagnostic methods (microscopy, RDT, and PCR) for the detection of malaria parasites in representative samples from Equatorial Guinea 11 Medical and Health Sciences 1108 Medical Microbiology. Malar. J..

[29] Edwards HM (2015). Novel cross-border approaches to optimise identification of asymptomatic and artemisinin-resistant *Plasmodium* infection in mobile populations crossing cambodian borders. PLoS ONE.

[30] Endeshaw T (2010). Comparison of Parascreen Pan/Pf, Paracheck Pf and light microscopy for detection of malaria among febrile patients, Northwest Ethiopia. Trans. R. Soc. Trop. Med. Hyg..

[31] Feleke DG, Tarko S, Hadush H (2017). Performance comparison of CareStart^TM^ HRP2/pLDH combo rapid malaria test with light microscopy in north-western Tigray, Ethiopia: a cross-sectional study. BMC Infect. Dis..

[32] Haanshuus CG (2016). A high malaria prevalence identified by PCR among patients with acute undifferentiated fever in India. PLoS ONE.

[33] Imwong M (2015). The epidemiology of subclinical malaria infections in South-East Asia: findings from cross-sectional surveys in Thailand-Myanmar border areas, Cambodia, and Vietnam. Malar. J..

[34] Iqbal J, Hira PR, Sher A, Aziz Al-Enezi A (2001). Diagnosis of imported malaria by Plasmodium lactate dehydrogenase (pLDH) and histidine-rich protein 2 (PfHRP-2)-based immunocapture assays. Am. J. Trop. Med. Hyg..

[35] Jahan F (2019). Malaria epidemiology and comparative reliability of diagnostic tools in Bannu; an endemic malaria focus in south of Khyber Pakhtunkhwa Pakistan. Pathog. Glob. Health.

[36] Kim SH (2008). Evaluation of a rapid diagnostic test specific for *Plasmodium vivax*. Trop. Med. Int. Health.

[37] Li P (2016). *Plasmodium malariae* and *Plasmodium ovale* infections in the China-Myanmar border area. Malar. J..

[38] Moges B (2012). Comparison of CareStart (TM) HRP2/pLDH COMBO rapid malaria test with light microscopy in north-west Ethiopia. Malar. J..

[39] Ranjan P, Ghoshal U (2016). Utility of nested polymerase chain reaction over the microscopy and immuno-chromatographic test in the detection of Plasmodium species and their clinical spectrum. Parasitol. Res..

[40] Ratnawati HM, Smits HL (2008). Point-of-care testing for malaria outbreak management. Trans. R. Soc. Trop. Med. Hyg..

[41] Richter J, Göbels K, Müller-Stöver I, Hoppenheit B, Häussinger D (2004). Co-reactivity of plasmodial histidine-rich protein 2 and aldolase on a combined immuno-chromographic-malaria dipstick (ICT) as a potential semi-quantitative marker of high *Plasmodium falciparum* parasitaemia. Parasitol. Res..

[42] Woyessa A, Deressa W, Ali A, Lindtjørn B (2013). Evaluation of CareStart^TM^ malaria Pf/Pv combo test for *Plasmodium falciparum* and *Plasmodium vivax* malaria diagnosis in Butajira area, south-central Ethiopia. Malar. J..

[43] Yan J (2013). Performance of two rapid diagnostic tests for malaria diagnosis at the China-Myanmar border area. Malar. J..

[44] Mehlotra RK (2019). Plasmodium falciparum parasitemia and band sensitivity of the SD Bioline Malaria Ag P.f/Pan rapid diagnostic test in Madagascar. Am. J. Trop. Med. Hyg..

[45] Getnet G (2015). Diagnostic performance of rapid diagnostic tests for the diagnosis of malaria at public health facilities in north-west Ethiopia. Trop. Med. Int. Health..

[46] Iqbal J, Khalid N, Hira PR (2002). Comparison of two commercial assays with expert microscopy for confirmation of symptomatically diagnosed malaria. J. Clin. Microbiol..

[47] Khorashad AS, Roshanravan M (2014). The comparison of microscopic method and rapid diagnostic test in detecting *Plasmodium* species. Int. J. Infect..

[48] Chanie M, Erko B, Animut A, Legesse M (2011). Performance of CareStart^TM^ Malaria Pf/Pv Combo test for the diagnosis of *Plasmodium falciparum* and *Plasmodium vivax* infections in the Afar Region, North East Ethiopia. Ethiop. J. Health Dev..

[49] Meena M (2009). Accuracy of a multispecies rapid diagnostic test kit for detection of malarial parasite at the point of care in a low endemicity region. Trans. R. Soc. Trop. Med. Hyg..

[50] Sharew B (2009). Evaluation of the performance of CareStart Malaria Pf/Pv combo and paracheck Pf tests for the diagnosis of malaria in Wondo Genet, southern Ethiopia. Acta Trop..

[51] van den Broek I (2006). Evaluation of three rapid tests for diagnosis of *P. falciparum* and *P. vivax* malaria in Colombia. Am. J. Trop. Med. Hyg..

[52] WHO. *M*alaria rapid diagnostic test performance. Results of WHO product testing of malaria RDTs : round 1 (2008), https://www.who.int/malaria/publications/atoz/9789241598071/en/ (2009).

[53] Maltha J (2010). Malaria rapid diagnostic tests: *Plasmodium falciparum* infections with high parasite densities may generate false positive *Plasmodium vivax* pLDH lines. Malar. J..

[54] Dondorp AM (2005). Estimation of the total parasite biomass in acute falciparum malaria from plasma PfHRP2. PLoS Med.

[55] Bell DR, Wilson DW, Martin LB (2005). False-positive results of a *Plasmodium falciparum* histidine-rich protein 2-detecting malaria rapid diagnostic test due to high sensitivity in a community with fluctuating low parasite density. Am. J. Trop. Med. Hyg..

[56] WHO. Guidelines for the treatment of malaria, https://www.who.int/malaria/publications/atoz/9789241549127/en/ (2015).

[57] Snounou G, Viriyakosol S, Jarra W, Thaithong S, Brown KN (1993). Identification of the four human malaria parasite species in field samples by the polymerase chain reaction and detection of a high prevalence of mixed infections. Mol. Biochem. Parasitol..

[58] Laurent A (2010). Performance of HRP-2 based rapid diagnostic test for malaria and its variation with age in an area of intense malaria transmission in southern Tanzania. Malar. J..

[59] Abeku TA (2008). Determinants of the accuracy of rapid diagnostic tests in malaria case management: evidence from low and moderate transmission settings in the East African highlands. Malar. J..

[60] Ebrahimzadeh A, Fouladi B, Fazaeli A (2007). High rate of detection of mixed infections of *Plasmodium vivax* and *Plasmodium falciparum* in South-East of Iran, using nested PCR. Parasitol Int.

[61] Zakeri S (2010). Detection of mixed *Plasmodium falciparum* and *P. vivax* infections by nested-PCR in Pakistan, Iran & Afghanistan. Indian J Med Res.

[62] Romay-Barja M (2015). Rural-Urban Differences in household treatment-seeking behaviour for suspected malaria in children at Bata district Equatorial Guinea. PLoS ONE.

[63] WHO. Malaria rapid diagnostic test performance. Results of WHO product testing of malaria RDTs: round 8 (2016–2018), https://www.who.int/malaria/publications/atoz/9789241514965/en/ (2018).

