# Supplementary figures and images for: Summary of discordant results between rapid diagnosis tests, microscopy, and polymerase chain reaction for detecting Plasmodium mixed infection: a systematic review and meta-analysis
Source: Sci Rep. 2020 Jul 29;10:12765. doi: 10.1038/s41598-020-69647-y (PMC7392751; doi:10.1038/s41598-020-69647-y)

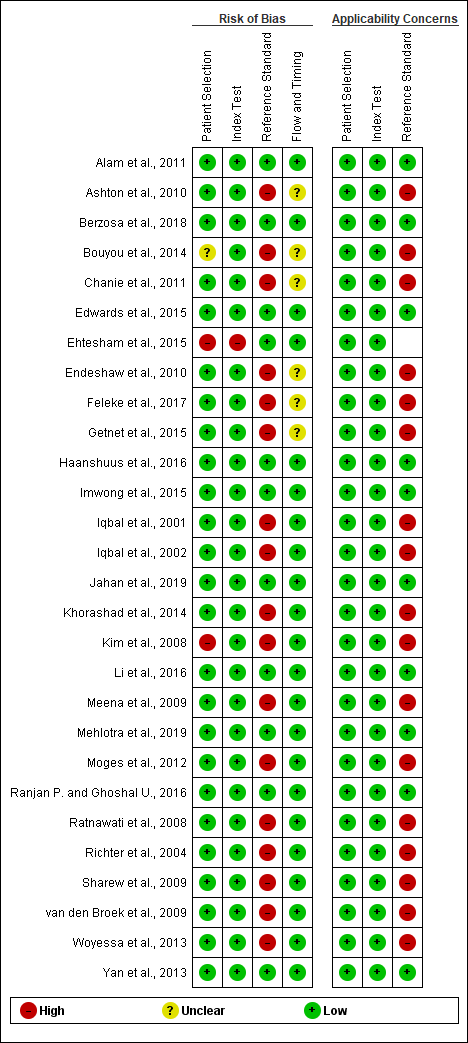

Supplement: Supplementary file 1 [file 41598_2020_69647_MOESM1_ESM.png]
