# Supplementary material for: Summary of discordant results between rapid diagnosis tests, microscopy, and polymerase chain reaction for detecting Plasmodium mixed infection: a systematic review and meta-analysis
Source: Sci Rep. 2020 Jul 29;10:12765. doi: 10.1038/s41598-020-69647-y (PMC7392751; doi:10.1038/s41598-020-69647-y)
Supplement: Supplementary file 2 — Supplementary Table S1. [file 41598_2020_69647_MOESM2_ESM.docx]

**Summary of discordant results between rapid diagnosis tests, microscopy, and polymerase chain reaction for detecting *Plasmodium* mixed-infection: A systematic review and meta-analysis**

Manas Kotepui^1*^, Kwuntida Uthaisar Kotepui^1^, Giovanni De Jesus Milanez^2^, Frederick Ramirez Masangkay^2^

^1^Medical Technology, School of Allied Health Sciences, Walailak University, Thasala, Nakhon Si Thammarat, Thailand

^2^Department of Medical Technology, Institute of Arts and Sciences, Far Eastern University-Manila, Manila, Philippines

**Table S1**

**Search terms**

("rapid diagnostic test"[All Fields] OR RDT[All Fields] OR dipstick[All Fields] OR ("chromatography, affinity"[MeSH Terms] OR ("chromatography"[All Fields] AND "affinity"[All Fields]) OR "affinity chromatography"[All Fields] OR "immunochromatography"[All Fields]) OR "rapid malaria antigen test"[All Fields]) AND (("malaria"[MeSH Terms] OR "malaria"[All Fields]) OR ("plasmodium"[MeSH Terms] OR "plasmodium"[All Fields])) AND ("mixed infection"[All Fields] OR "co-infection"[All Fields] OR ("coinfection"[MeSH Terms] OR "coinfection"[All Fields]))
