# Supplementary material for: Summary of discordant results between rapid diagnosis tests, microscopy, and polymerase chain reaction for detecting Plasmodium mixed infection: a systematic review and meta-analysis
Source: Sci Rep. 2020 Jul 29;10:12765. doi: 10.1038/s41598-020-69647-y (PMC7392751; doi:10.1038/s41598-020-69647-y)
Supplement: Supplementary file 3 — Supplementary Table S2. [file 41598_2020_69647_MOESM3_ESM.docx]

**Table S2 Egger’s test**

| Std_Eff | Coef. | Std. Err. | t | P>\|t\| | [95% Conf. Interval] |
| --- | --- | --- | --- | --- | --- |
| slope | -2.606923 | 8.005815 | -0.33 | 0.747 | -18.95698-13.74313 |
| bias | 25.2071 | 17.74693 | 1.42 | 0.166 | -11.03697-61.45117 |
